# E as in Enigma: The Mysterious Role of the Voltage-Dependent Anion Channel Glutamate E73

**DOI:** 10.3390/ijms24010269

**Published:** 2022-12-23

**Authors:** Alexander Bernhard Rister, Thomas Gudermann, Johann Schredelseker

**Affiliations:** 1 Walther Straub Institute of Pharmacology and Toxicology, Faculty of Medicine, LMU Munich, 80336 Munich, Germany; 2 Deutsches Zentrum für Herz-Kreislauf-Forschung, Partner Site Munich Heart Alliance, Munich, Germany

**Keywords:** VDAC, glutamate 73, mitochondria, hexokinase, cholesterol

## Abstract

The voltage-dependent anion channel (VDAC) is the main passageway for ions and metabolites over the outer mitochondrial membrane. It was associated with many physiological processes, including apoptosis and modulation of intracellular Ca^2+^ signaling. The protein is formed by a barrel of 19 beta-sheets with an N-terminal helix lining the inner pore. Despite its large diameter, the channel can change its selectivity for ions and metabolites based on its open state to regulate transport into and out of mitochondria. VDAC was shown to be regulated by a variety of cellular factors and molecular partners including proteins, lipids and ions. Although the physiological importance of many of these modulatory effects are well described, the binding sites for molecular partners are still largely unknown. The highly symmetrical and sleek structure of the channel makes predictions of functional moieties difficult. However, one residue repeatedly sticks out when reviewing VDAC literature. A glutamate at position 73 (E73) located on the outside of the channel facing the hydrophobic membrane environment was repeatedly proposed to be involved in channel regulation on multiple levels. Here, we review the distinct hypothesized roles of E73 and summarize the open questions around this mysterious residue.

## 1. Introduction

The voltage-dependent anion channel (VDAC) is a 32 kDa pore-forming protein in the outer mitochondrial membrane, composed of 283 to 294 amino acids depending on the respective isoform. It is the main passageway over the outer mitochondrial membrane and conducts ions and small metabolites into and out of mitochondria. Higher organisms express 3 isoforms, VDAC1-3, whose distinct roles are still under debate. While VDAC1, as the best researched isoform, was described to be a major regulator of apoptosis and a regulator of cellular energetics by conducting ATP, the contribution of VDAC2 and 3 to these processes are less clear. Both VDAC1 and VDAC2 were described to be regulators of mitochondrial Ca^2+^ and to interact with the Ca^2+^ release channels of other cellular organelles, such as lysosomes [1] or the endoplasmic/sarcoplasmic reticulum [2,3]. VDAC3 was recently reported to have a major role in cellular ROS signaling [4].

**Figure 1:**
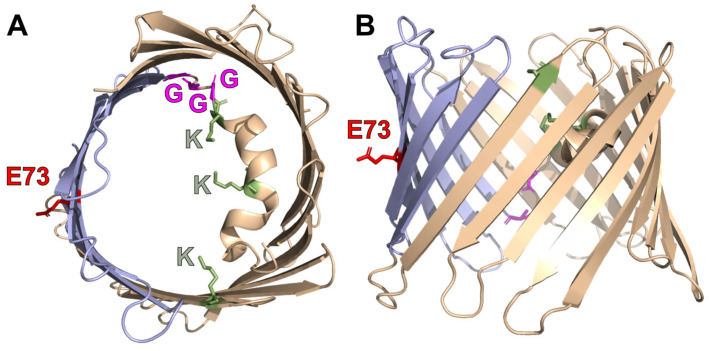
Structure of VDAC and localization of glutamate 73. Top (**A**) and side view (**B**) of the voltage dependent anion channel (VDAC1, pdb: 3 emn) as cartoon representation. Glutamate 73 is shown in red. A six beta sheet region around E73 (purple) was suggested to show higher protein dynamics compared to the rest of the barrel and to interact with the opposite helix to control ion permeation. A glycine stretch previously suggested to mediate protein interaction is shown in magenta and lysines coordinating ATP permeation are shown in green.

Although high resolution structures are so far only available for VDAC1 [5] and VDAC2 [6], it can be assumed that all three isoforms share high structural similarity. VDAC proteins are composed of 19 β-sheets connected by small linkers and arranged in an anti-parallel orientation to form a large barrel in the membrane. The inside of the barrel is lined by an N-terminal α-helix (Figure 1). Because of their large pore diameter of approximately 1.5–2 nm, VDACs are often described to be freely permeable to ions and small metabolites. However, accumulating evidence contradicts this idea and describes multiple modes of regulation for VDAC permeability [7,8]. These include subcellular localization, interaction with partner proteins, and most importantly, the gating of the channel.

When inserted into planar lipid bilayers, VDAC resides in an anion-selective, 4nS high-conductance state at potentials around 0 mV, shows vigorous gating behavior at test potentials around ±20–40 mV and resides in several more cation selective low-conductance states (around 2 nS) at potential larger then 50 mV or lower then −50 mV [9,10,11]. Although it still remains unclear whether the membrane potential across the outer mitochondrial membrane is large enough to serve as the physiological trigger for channel gating, this gating behavior was often linked to channel activity and regulation.

Gating of the channel was shown to be influenced by various factors, such as the lipidic environment [12,13,14], PKA phosphorylation [15], the local Ca^2+^ concentration [16] or the presence of VDAC binding partners like hexokinase [17,18] or members of the Bcl-2 family [19]. Physiological experiments, mainly on cultured or freshly isolated cells, further indicate a substantial regulation of the channel, which is highly relevant for its role in apoptosis or Ca^2+^ signaling.

Despite several reports about regulation of the channel through the aforementioned factors, the molecular underpinnings still remain largely elusive, especially because the highly symmetrical and very sleek structure of VDAC makes identification of functionally relevant moieties in the channel difficult. Most reports focus on the N-terminal domain, including the helix as a relevant structure for channel gating and interaction with other proteins. As such, a lysine-rich motif in the helix was suggested to guide ATP permeation [20], while a glycine rich element in the distal part of the helix was shown to mediate binding to partner proteins [21] (Figure 1).

On the other hand, motifs in the barrel itself are not well characterized, with the exception of one prominent amino acid, a glutamate at position 73 (E73). This glutamate was first described in 1993 as an important residue for the binding of hexokinase [22]. In further publications, E73 was suggested to be involved in Ca^2+^ binding and permeation [23], lipid binding [24,25], and channel dimerization [26]. However, looking at the molecular structure of VDAC, E73 is largely buried inside the membrane, making functional models very difficult. In the following, we will discuss proposed roles of E73 in channel function and regulation.

## 2. Intrinsic Channel Function

Looking at the high-resolution structure of VDAC, E73 is located in the fourth β-sheet of the transmembrane barrel with its side chain located on the outside of the barrel and sticking into the middle plane of the hydrophobic membrane environment (Figure 1). The area around E73 is located opposite of the N-terminal helix, which was proposed to enhance barrel stability through interaction with the barrel. Consequently, the area around E73 is believed to be more flexible due to the lack of the stabilizing effect of the helix, which is in line with b-factors for this area obtained from the crystal structure [27].

This flexibility was proposed to be, at least in part, also induced by E73: molecular dynamics simulations revealed that the charge of E73 enhances protein dynamics of the area comprising the first four to six β-sheets of the barrel (with E73 being located in the fourth β-sheet) [28]. Rendering E73 uncharged by protonation or mutation of E73 to glutamine (E73Q) or valine (E73V) dramatically reduced barrel dynamics. From these experiments, a gating mechanism involving large deformations of the barrel from a round towards a more elliptical state was proposed, with the area around E73 as a critical element of this movement (Table 1). Indeed, NMR spectroscopy of the VDAC1 E73V mutant revealed a more elliptical barrel compared to the available wild-type structures [29,30]. This model is further supported, by the idea that interaction of negative amino acids in the β-sheets around E73 interact with the helix to determine ion selectivity [20,31,32].

However, in later experiments performed in planar lipid bilayers, the exchange of E73 for glutamine or alanine revealed no changes in the voltage-induced gating of recombinant VDAC1 [13], contradicting the hypothesis of E73 being a critical element of channel gating (Table 1). It is still questionable though, if the channel is present in its apo-form under physiological conditions, or if an association with molecular partners, which is absent in planar lipid bilayers is required to unlock the E73 effect in vivo. In this regard, the molecular dynamics experiments also proposed that in addition to the enhanced protein dynamics the charge at E73 would induce a thinning of the membrane in this area [28]. This was interpreted as a sign for the regulation of the channel through interaction with other molecules such as ions, lipids or proteins. Indeed, another series of simulation experiments [33] showed a distortion of the channel in response to voltage by the rearrangement of charged residues in the β-sheets around E73 that was attributed to membrane thinning. In this study, sodium ions were repeatedly observed near E73 in the open state, indicative of a higher accessibility of this residue to charged molecules. It was postulated that the accessibility of E73 to molecular binding partners induced by thinning of the membrane would stabilize the channel in the open state.

One potential binding partner could be the channel itself; several lines of evidence indicate that dimerization of VDAC serves as a mode of channel regulation in apoptosis and metabolic control [34,35]. Bergdoll et al. reported a critical role for an interaction of E73 with S43 (β-sheet 2) in channel dimerization, which is dependent on its protonation state [26]. The formation of a VDAC dimer was reported at low pH, which was largely abolished by mutation of E73 to alanine or glutamine.

Taken together, it appears unlikely that E73 can influence channel gating in its apo-state (Table 1), but dimerization or interaction with other protein partners are required to unlock E73 effects. A higher accessibility of E73 to these binding partners is achieved by a higher degree of flexibility of the area around E73. In the following, we will review the most important findings about the proposed E73 ligands: calcium, lipids and proteins.

## 3. Calcium

One of the most researched interaction partners of VDAC is Ca^2+^. Mitochondria can take up vast amounts of Ca^2+^ and the uptake and release of Ca^2+^ from mitochondria is a highly regulated process. Disturbances in mitochondrial Ca^2+^ uptake are associated with human diseases such as cardiac, metabolic and neurological diseases [36,37], as well as cancer [38,39], and consequently mitochondrial Ca^2+^ uptake was proposed to be a promising pharmacological target structure [40,41].

While in the inner mitochondrial membrane, the mitochondrial calcium uniporter complex (MCUC) is the main uptake route for Ca^2+^, VDAC is the main passageway for Ca^2+^ over the OMM, and several reports suggest a regulation of mitochondrial Ca^2+^ uptake at this stage [8,42]. Specifically, a critical role for VDAC and E73 was reported for local Ca^2+^ transfer from cellular organelles into mitochondria. A tight contact between lysosomes and mitochondria exists and disruption of this cross-talk was shown to be associated with lysosomal storage diseases. The direct coupling is essential for the transfer of Ca^2+^ from lysosomes into mitochondria via a TRPML1-VDAC1 axis [1]. Interestingly, this direct shuttling was critically dependent on the presence of E73 in VDAC1, and was completely abolished when E73 was mutated to glutamine.

Similarly, in cardiomyocytes, mitochondria form close contact sites with the sarcoplasmic reticulum, the main cellular Ca^2+^ store. This network involves a very close interaction between the SR Ca^2+^ release channel, the ryanodine receptor (RyR) and VDAC2 [2,43] and facilitates a local mitochondrial Ca^2+^ uptake near the SR Ca^2+^ release sites. This mechanism was shown to modulate intracellular Ca^2+^ signals during excitation-contraction coupling, and to buffer erratic Ca^2+^ release during diastole to prevent arrhythmogenesis [44,45]. Interestingly, E73 was critically required to mediate Ca^2+^ transfer between the SR and mitochondria in cardiomyocytes and as such to be protective against arrhythmia [46]. The expression of wild-type VDAC2 but not a mutant in which E73 was mutated to glutamine mediated SR-mitochondria Ca^2+^ transfer in cardiomyocytes, and the overexpression of wild-type VDAC2, but not E73Q, restored rhythmic cardiac contractions in a zebrafish arrhythmia model.

Two mechanisms are conceivable to be relevant for a regulation of Ca^2+^ flux through VDAC: on the one hand, VDAC was suggested to change its permeability for Ca^2+^ depending on its open state [47,48], while on the other hand, Ca^2+^ binding to the channel at concentrations larger then 600 nM was itself described to be a modulator of channel gating and thus Ca^2+^ permeation [16].

As outlined above, a direct role of E73 in channel gating appears unlikely, as lipid bilayer experiments demonstrated that E73 is not involved in the regulation of channel gating, at least not for monomers of the apo-channel [13]. Furthermore, E73 did not influence the ion selectivity of VDAC1 in lipid bilayers: while the channel is selective for anions in the open state, the closed state is less anion-selective and thus favors Ca^2+^ currents [47,48]. However, no differences in ion selectivity were observed between wild-type VDAC1 and the E73Q mutant, neither in the open state nor a blocked state that was induced by the addition of α-synuclein, a known blocker of VDAC1 [49] (Table 1). With the notion that E73 does not affect gating or selectivity of the apo-channel, a role of E73 in Ca^2+^-mediated modulation of the channel or a role in Ca^2+^-mediated interaction of the channel with regulatory partners appears most likely.

For both, a binding site for Ca^2+^ involving E73 must exist in the channel. After Gincel et al. had reported that VDAC transports Ca^2+^ into mitochondria and that this transport is sensitive to ruthenium red (RuR) [50], the same group reported in 2007 that Ca^2+^ competes with 25 µM azido ruthenium (AzRu) for a binding site involving E73 and E202 in a dose-dependent manner with a half-maximal effect at 100 µM [23]. The mutation of E73 to glutamine in VDAC1 eliminated AzRu sensitivity of VDAC in lipid bilayers and AzRu-photolabeling of the channel, and prevented an AzRu induced protection from apoptosis in T-REx-293 cells. Since the molecular structure of VDAC was unknown at the time, the authors postulated a binding site formed by the two residues. This model, however, does not hold true anymore after the molecular structure was resolved. In the commonly accepted molecular structure, which was solved in 2008 by two independent groups simultaneously through NMR and crystallography, respectively [5,51], E73 faces the lipidic environment of the membrane, raising the question how E73 could bind Ca^2+^ inside this hydrophobic environment. Ujwal et al. concluded that VDAC could only hold Ca^2+^ when two monomers of the channel assemble in an antiparallel dimer and only under very high concentrations of Ca^2+^, both of which are not expected to appear under physiological conditions [5].

In conclusion, a critical role of E73 can be observed for mitochondrial Ca^2+^ uptake, which is directly relevant for human diseases and the treatment thereof; however, the molecular underpinnings of the VDAC-Ca^2+^ interaction remain elusive. With the notion that E73 was described not to influence gating behavior or ion selectivity and the absence of a defined Ca^2+^ binding site involving E73, it remains unclear how E73 can control Ca^2+^ flux through VDAC at a molecular level (Table 1). A role of larger interaction partners, such as lipids or proteins, is most feasible.

## 4. Lipids

The lipidic environment, in which VDAC is embedded, was repeatedly shown to influence its physiology including voltage gating [12,13], ion selectivity [52] and interaction with partner proteins [53]. Beyond the idea that general changes in protein stability happen in response to the surrounding lipidic environment, several lipids were shown to have distinct binding sites on the channel, particularly sites including E73.

Ceramide is a sphingolipid, which can induce mitochondrial apoptosis and limit cancer cell proliferation by blocking cell cycle transition [54]. Through a chemical screening, VDAC 1 and 2 were found to be among its binding partners [55] and a structural constellation, in which the head group of the ceramide is bound to the negative charge of a glutamate, specifically E73, was proposed. Indeed, the mutation of E73 to Q in VDAC1 rendered colon cancer cells resistant to ceramide-induced apoptosis.

**Figure 2:**
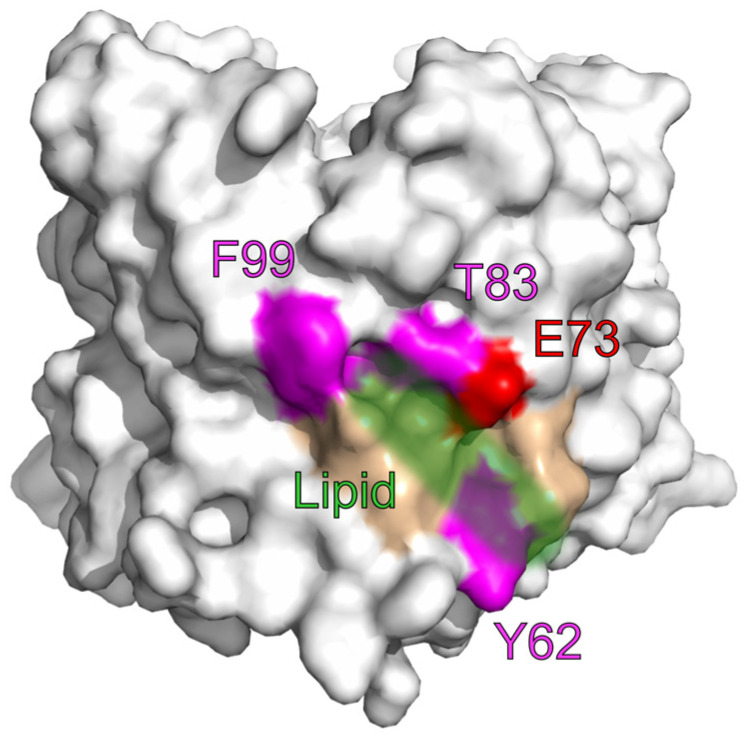
Proposed lipid binding pocket. Using a photo-affinity approach, a binding pocket around E73 (red) for cholesterol and allogenanolone was proposed by Cheng et al. [14]. Residues Y62, T83, F99 (magenta) and E73 (red) were photo labeled by sterol-based photolabeling reagents and labeling was competitively inhibited by cholesterol and allopregnanolone (lipid, green).

Among the most researched lipids that were shown to interact with VDAC are steroids, with cholesterol as the most prominent binding partner. Binding of cholesterol to VDAC was shown repeatedly [56,57] and cholesterol was shown to modulate VDACs oligomeric state and interaction with hexokinase [58]. However, also a vice versa effect was described in which a protein complex involving VDAC modulates the membrane cholesterol content [59,60]. Multiple binding sites were proposed by molecular docking [61] and NMR [51]. However, a direct investigation of the cholesterol-VDAC interaction domain only confirmed a binding site involving E73. A click chemistry approached, which allowed identification of protein-ligand interaction also in hydrophobic environments, revealed E73 as part of a binding site for neurosteroids in mouse VDAC1 [25]. This work is supported by the findings that sterol-based photolabeling reagents label residues Y62, T83, and F99 additionally to E73 forming a binding pocket around E73 (Figure 2), and that this labeling was competitively prevented using cholesterol and allopregnanolone in a concentration of 30 µM [14]. Furthermore, mutating E73 to Q, or A, lead to a significant decrease in the binding efficiency of cholesterol and allopregnanolone to VDAC1. Interestingly, a similar reduction was observed when the pH was lowered to 5–6 indicating that the charge at E73 is essential for the binding of lipids.

Interestingly, however, at least two independent reports have investigated the effect of cholesterol and allopregnanolone on VDAC in lipid bilayers and found no differences in channel gating using different concentrations of the lipid [13,14]. It is thus feasible that instead of directly modeling the channels biophysical properties, binding of cholesterol to E73 affects VDAC dimerization or its interaction with hexokinase.

## 5. Hexokinase

Hexokinases are enzymes which play a key role for glycolysis by phosphorylating glucose to glucose-6-phosphate. Hexokinases I and II are often found to be bound to mitochondria, which ensures availability of both glucose and ATP at the active center of the enzyme, delivered from the cytosol and mitochondria respectively [62]. In 1979, Felgner et al. identified a 31 kDa protein as the binding partner and anchor protein of hexokinase in the mitochondrial membrane [63], which was later identified as VDAC [64]. A particularly important role of this hexokinase-VDAC interaction induced accumulation of hexokinase at the outer mitochondrial membrane was described for cancer: cancer cells have a very high rate of glycolysis to match their energy demand. Consequently, the amount of hexokinase bound to VDAC was described to be elevated in these cells [65], and disruption of this interaction was suggested as an anti-tumor therapy [66]. In addition to the anchoring effect, hexokinase binding to VDAC was shown to induce channel closure and to be protective against apoptosis, securing cancer cell survival [67].

Therefore, a binding site for hexokinase on VDAC must exist. After the discovery that dicyclohexylcarbodiimide (DCCD) can compete out the VDAC-hexokinase interaction [68], De Pinto et al. used [^14^C]DCCD-labelling of VDAC followed by enzymatic digestion to identify the putative common VDAC-binding site of hexokinase and DCCP [22]. They found a direct binding of [^14^C]DCCD to E73. These experiments were confirmed using a series of assays performed with VDAC1 and a mutant of VDAC1 in which E73 was mutated to Q: overexpression of hexokinase prevented apoptosis induced by overexpression of wild-type VDAC1 but not E73Q and binding of 30–35 mU of hexokinase 1 to isolated yeast mitochondria was reduced by 70% in mitochondria expressing the E72Q mutant compared to wild-type VDAC1 [69] (Table 1). Furthermore, hexokinase did colocalize with mitochondria in cells expressing wild-type VDAC1 but not VDAC1 E73Q and finally, hexokinase reduced VDAC1 conductance in lipid bilayers, while currents through VDAC1 E73Q remained unaffected [17]. Experiments in which alkalization of the cell diminished hexokinase binding to mitochondria again indicate an important role of the protonation status of E73 for hexokinase binding, comparable to what was previously described for dimerization and cholesterol binding [70].

Coming back to the role of cholesterol on VDAC function, it is of note that an interplay of VDAC, cholesterol and hexokinase at E73 was described; as such, the membrane cholesterol content was shown to be enriched in carcinoma cells, which was associated with a reduction in oxidative phosphorylation. A protein complex involving VDAC was shown to be accountable for this process. The overexpression of VDAC E73Q in contrast increased oxidative phosphorylation and reduces membrane cholesterol content [59].

Taken together, the interaction of VDAC with hexokinase is essential for the regulation of the cellular metabolism, in particular the change from oxidative phosphorylation to enhanced glycolysis in cancer cells. This interaction is mediated by E73 and is maybe the most intensively researched interaction of VDAC with a molecular partner. A reduction in channel conductance together with downstream effects, such as a reduction of cholesterol content and protection against apoptosis, are associated with this interaction. However, most of these experiments were conducted using overexpression systems, and factors regulating the amount of hexokinase being bound to VDAC under physiological conditions are still unknown. The lipidic environment, intracellular pH, and cellular ions such as Ca^2+^ might be involved in this process.

**Table 1:** The role of E73 for VDAC channel functions. The passage of ions and small metabolites though VDACs is regulated by channel gating, selectivity of the pore and interaction with molecular partners. E73 was repeatedly suggested to be critically involved in these processes. A direct comparison of key experimental results is presented.

Molecular Process	Results in Favor of an Involvement of E73	Results Contradicting a Direct Involvement of E73
Voltage-gating	- In molecular dynamics simulations E73 enhances barrel flexibility to allow transition to a more elliptical state [29,30].	- Replacing E73 by Q did not alter voltage gating in lipid bilayers [13].
Selectivity	- The flexible area around E73 was suggested to interact with the helix to determine ion selectivity [20,31,32].	- No differences in ion selectivity were observed in lipid bilayers upon exchange of E73 for Q [49].
Modulation of channel function through interaction (scaffolding)	- In simulation experiments the charge E73 induces a thinning of the membrane to facilitate molecular interactions [28,33]. - Experimental data shows reduced formation of dimers [26], as well as binding of lipids [14] and hexokinase [69] for E73Q mutants.	- The molecular structure reveals a location of E73 within the membrane making it poorly accessible [5,6].

## 6. Conclusions

**Figure 3:**
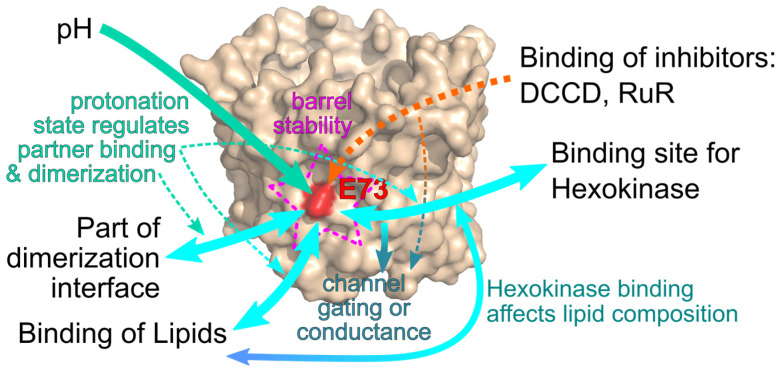
Interactions at E73. Multiple interactions of molecular partners were described for E73. The charge at E73 renders the protein more flexible to allow ions and binding partners to access E73 (magenta). The protonation state of E73 is affected by the intracelullar pH (green) and influences channel dimerization and the binding of lipids and hexokinase to the channel (light blue). These interactions affect channel gating and conduction but could also influence each other, for example through a hexokinase induced change in membrane lipid composition. Synthetic channel modifiers such as RuR or DCCD also bind to E73 and directly affect channel gating and conductance (orange).

Taken together, multiple interactions of VDAC with molecular partners were described to occur around E73. It is conceivable that these mechanisms do not act independently of each other, but a complex interplay between these factors adapts VDAC function to the current state of the cell (Figure 3). In this instance, the protonation state of E73 was identified as a central factor for many of those mechanisms. An increased conformational flexibility of the VDAC barrel around E73 makes this buried area more accessible to ions including protons. Through this mechanism, changes in intracellular pH can influence VDAC dimerization, the binding of hexokinase, as well as the binding of certain lipids, such as cholesterol or neurosteroids, to the channel. The binding of lipids was shown to influence hexokinase interaction and the binding of hexokinase was shown to influence channel gating and conductance. Furthermore, a feedback mechanism appears to exist through which binding of hexokinase can influence the lipidic membrane composition, in particular cholesterol content.

This complex interplay of regulatory mechanisms allows VDACs to adapt to changes in the metabolic state of the cell mediated by multiple layers of regulation and fine-tuning. However, many unanswered questions within this network are still open and E73 still remains to be one of the big enigmas of VDAC.
